# Food origin influences microbiota and stable isotope enrichment profiles of cold-adapted Collembola (*Desoria ruseki*)

**DOI:** 10.3389/fmicb.2022.1030429

**Published:** 2022-11-24

**Authors:** Cao Hao, Nadieh de Jonge, Dong Zhu, Lichao Feng, Bing Zhang, Ting-Wen Chen, Donghui Wu, Jeppe Lund Nielsen

**Affiliations:** ^1^State Environmental Protection Key Laboratory of Wetland Ecology and Vegetation Restoration, School of Environment, Northeast Normal University, Changchun, China; ^2^Key Laboratory of Wetland Ecology and Environment, Northeast Institute of Geography and Agroecology, Chinese Academy of Sciences, Changchun, China; ^3^Department of Chemistry and Bioscience, Aalborg University, Aalborg, Denmark; ^4^Key Laboratory of Urban Environment and Health, Ningbo Urban Environment Observation and Research Station, Institute of Urban Environment, Chinese Academy of Sciences, Xiamen, China; ^5^Forest Protection, Beihua University, Jilin, China; ^6^Key Laboratory for Earth Surface Processes of the Ministry of Education, Institute of Ecology, College of Urban and Environmental Science, Peking University, Beijing, China; ^7^Biology Centre of the Czech Academy of Sciences, Institute of Soil Biology and Biogeochemistry, České Budějovice, Czechia; ^8^Key Laboratory of Vegetation Ecology, Ministry of Education, Northeast Normal University, Changchun, China; ^9^Jilin Provincial Key Laboratory of Animal Resource Conservation and Utilization, Northeast Normal University, Changchun, China

**Keywords:** feeding ecology, host-microbiota interaction, soil arthropods, springtail, stable isotope, winter biodiversity

## Abstract

Collembola are a group of globally distributed microarthropods that can tolerate low temperature and are active in extremely cold environments. While it is well known that animal diets can shape their microbiota, the microbiota of soil animals is not well described, particularly for animals with limited food resources, such as Collembola active in winter at low temperatures. In this study, we explored the effects of three different food sources; corn litter (agriculture grain residuals), Mongolian oak litter (natural plant residuals), and yeast (common food for Collembola culture), on the microbiota of a winter-active Collembola species, *Desoria ruseki*. We found that microbial diversity and community composition of the Collembola were strongly altered after feeding with different food sources for 30 days. Collembola individuals fed on corn litter harbored the highest bacterial richness and were dominated by a representative of *Microbacteriaceae*. In contrast, those fed on yeast exhibited the lowest bacterial richness and were primarily colonized by *Pseudomonas*. The microbial communities associated with the winter-active Collembola differed significantly from those observed in the food. Collembola nutrient turnover also differed when cultured with different food sources, as indicated by the C and N stable isotopic signatures. Our study highlights microbial associations with stable isotopic enrichments of the host. Specifically, the *Arthrobacter* was positively correlated with δ^13^C enrichment in the host. Representatives of *Microbacteriaceae*, *Micrococcaceae*, TM7a, *Devosia*, and *Rathayibacter* were positively correlated with δ^15^N enrichment of the host. Our study indicates that food sources are major determinants for Collembola microbiota that simultaneously alter consumers’ isotopic niches, thereby improving our understanding of the roles played by host-microbiota interactions in sustaining soil biodiversity during the winter.

## Introduction

Soil arthropods can tolerate cold temperatures and maintain their activities in snowy and icy environments, especially at high latitudes of the northern hemisphere (Hågvar, 2010; Zhang et al., 2014; Leo et al., 2021). Animal-associated microbiota provides important functions for the host in resource utilization, energy metabolism, and pathogen defense (Engel and Moran, 2013; Bahrndorff et al., 2018). Symbiotic microbes in termites facilitate digestion of lignocellulose and mineralize nitrogen-rich humus components, thereby playing an important role in soil carbon and nitrogen cycling (Bashir et al., 2013; Brune, 2014). Moreover, the gut microbiota can contribute to nutrient turnover and energy orchestration of the animal host (Chevalier et al., 2015; Xiang et al., 2019a; Sepulveda and Moeller, 2020). Previous studies have focused on understanding symbiotic microbiota and their ecological functions in invertebrates, revealing that diet composition may shape the microbiota of the host (Mikaelyan et al., 2015; Xiang et al., 2019a; Kennedy et al., 2020). The diversity and composition of microbial communities in the Collembola gut have been shown to be affected by changes in diet, which can in turn affect host development and health (Xiang et al., 2019a). Diet influences on the microbiota associated with soil invertebrates, such as earthworms, beetles, termites, and Collembola, have primarily been studied in the growing season (Mikaelyan et al., 2015; Kudo et al., 2019; Xiang et al., 2019a; Hu et al., 2020). However, since food source availability is more limited in winter periods, as compared to the growing season, whether food resources affect the microbiota associated with cold-adapted soil invertebrates has yet to be explored.

Cold-adapted Collembola can live in harsh snowy microhabitats under the snow (subnivean), in the snow (intranivean), or on the snow surface (supranivean; Hågvar, 2010; Zhang et al., 2014). Members of the genus *Desoria* can be active in cold regions with high density in winter (Zhang et al., 2017; Hao et al., 2020; Valle et al., 2021). The species *Desoria ruseki* is widely distributed in diverse snow-covered habitats, including forestland and cropland. However, disturbance due to anthropogenic land-use change may force Collembola to feed on different litter residuals and/or microbiota from the environments (Oliver and Morecroft, 2014; Susanti et al., 2021), even though they are usually generalist feeders (Potapov et al., 2021). Cold-adapted Collembola are also likely to consume a great variety of food sources such as litter and environmental microorganisms (Zettel et al., 2002; Bokhorst and Wardle, 2014). In cropland, corn is one of potential resources for Collembola (Liu et al., 2012; Scheunemann et al., 2015); the corn residuals can provide habitats and food. In forestland, natural residuals of plants such as the Mongolian oak constitute an important food resource for Collembola (Sadaka-Laulan and Ponge, 2000; Zhang et al., 2021). In contrast, yeast has been frequently used as a common food source for Collembola cultures (e.g., Buse et al., 2013; Xiang et al., 2019a). It can therefore be hypothesized that the microbiota of cultivated Collembola could be impacted by different resources from habitats, since consumers’ dietary changes can regulate the microbiota associated with them (Knapp et al., 2009; Xiang et al., 2019a). The microbiota of Collembola has been suggested to be shaped by host diet in growing seasons (Xiang et al., 2019a); however, for cold-adapted Collembola with limited food resources and low temperature, this microbiota association remains unexplored.

Carbon and nitrogen stable isotope fractionation (δ^13^C and δ^15^N) are useful indicators of host feeding habits and trophic position (Potapov et al., 2019), indirectly reflecting the variations of nutrient turnover in animal tissue (Cherel et al., 2005; Ding et al., 2020). The consumer is enriched in heavy nitrogen (^15^N) relative to food (Potapov et al., 2021). In contrast, the content of ^13^C changes little from food source to consumer and can be used to trace the carbon sources used by the consumer (Potapov et al., 2019). Recently published studies have shown that the microbiota associated with soil animals plays an important role in the isotopic enrichment of the host (Zhu et al., 2018; Xiang et al., 2019a). For example, the ingestion of microplastics altered the δ^13^C and δ^15^N compositions in Collembola tissues because microplastics induced the change in gut microbes, further mediating the turnover of carbon and nitrogen in the Collembola body (Zhu et al., 2018; Xiang et al., 2019b). It was found that the richness of the gut microbiota of *Folsomia candida* related to different diets was positively correlated with δ^15^N accumulations in host tissue, suggesting that a diverse gut microbial community enhances the nitrogen turnover rate (Xiang et al., 2019a). However, no previous studies have examined changes in stable isotopic enrichment in cold-adapted soil animals after ingestion of different food resources. The relationship between changes in microbiota of soil invertebrates active in cold winter and changes in host tissue isotopic fractionation during this season is also poorly understood.

In this study, we assessed the microbiota associated with the cold-adapted Collembola species, *Desoria ruseki*, from a manipulation experiment provided with different food sources: corn litter, Mongolian oak litter, and yeast using high throughput sequencing of the bacterial 16S rRNA gene. In addition, the carbon and nitrogen stable isotopic compositions of the cold-adapted Collembola populations and their foods were analyzed to indicate host trophic niche and nutrient turnover. The objectives were to determine whether different food sources affect the microbiota of cold-adapted Collembola and evaluate the effect of different food sources on δ^13^C and δ^15^N enrichments, in order to explore how the food source and microbial changes alter the isotopic enrichment of Collembola. These findings will improve our understanding of the potential impacts of food resource changes (e.g., due to land-use change) on the microbial community of generalist feeders and extend our knowledge on best laboratory practices to study host-microbiota interactions of active soil fauna in cold wintertime.

## Materials and methods

### Collembola cultures

The epedaphic cold-adapted Collembola species, *Desoria ruseki* used in the present study was collected by pitfall traps (Hao et al., 2020) from a forest plantation of larch in Huanxi town (43°48′15″N, 126°25′40″E) in Jilin city, Jilin province, China in March 2021. The pitfall traps were installed in the snow layer with the upper rim of the container level with the snow surface. The air temperature of daytime during the sampling period was 0°C–5°C. After the Collembola were transported to the laboratory, they were subjected to an acclimation period of a week in the lab. Collembola were identified based on morphological traits. All Collembola were cultured in plastic vessels floored with 8:1 gypsum and charcoal mixture in manipulation trial. The Collembola were kept at 5°C with a 12:12 h dark/light period according to the phenology of the sampling region and were cultured for 30 days. All Collembola used for the food manipulation experiment were development-synchronized *Desoria ruseki* adults, which were starved for 3 days before food manipulation to avoid carry over effects based on different feeding regimes of the indigenous populations. We observed few Collembola eggs, but none of these did successfully hatch under these growth conditions.

### Experimental design and food resources

**Figure 1:**
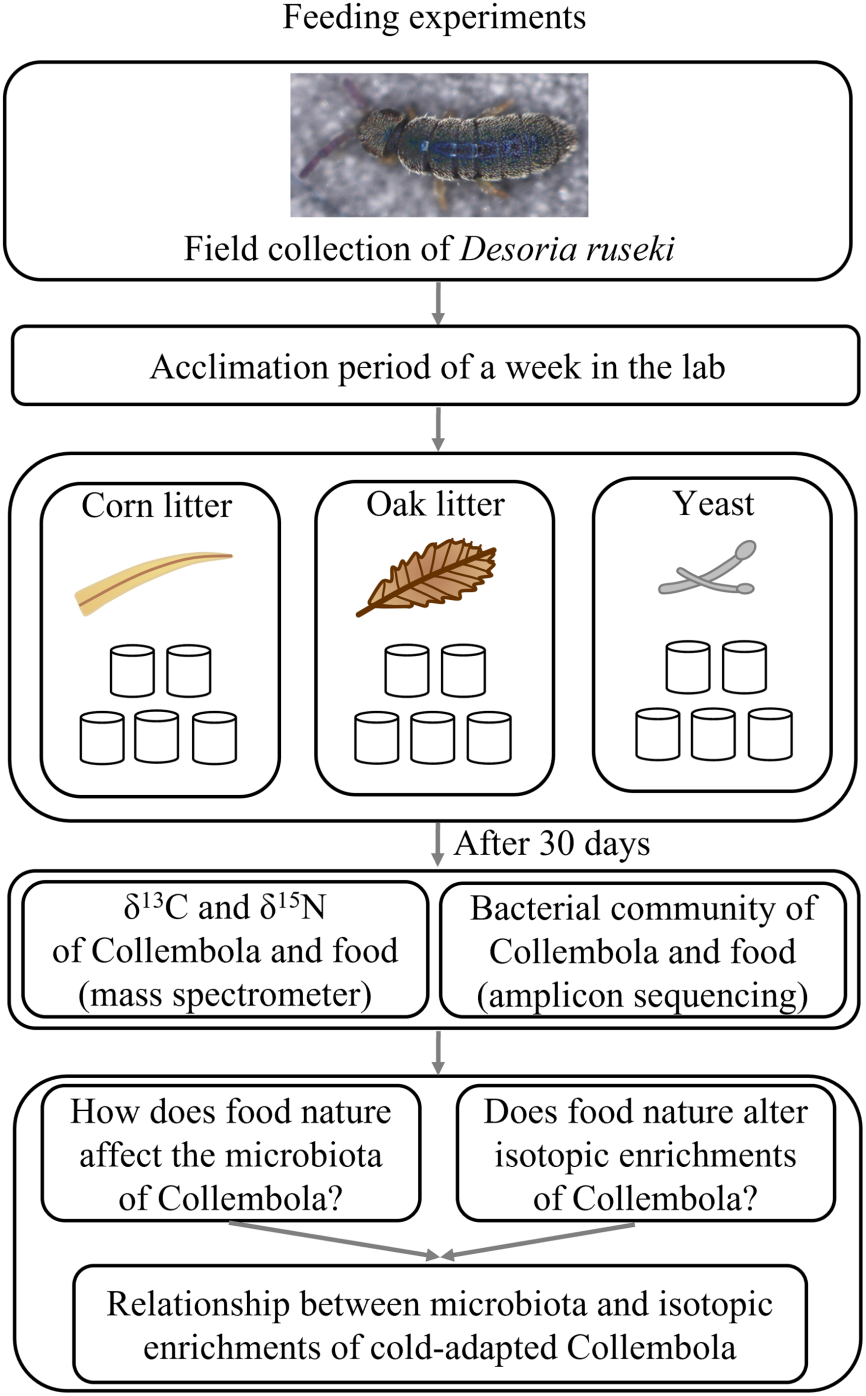
Schematic representation of the experimental setup.

Three food resources were used to feed Collembola in this study: (1) Corn litter (fallen leaves from *Zea mays*); (2) Mongolian oak litter (fallen leaves of *Quercus mongolica* Fisch. ex Ledeb.); (3) Yeast (*Saccharomyces cerevisiae* of commercial source; the common food for Collembola culture; Figure 1). Corn litter and Mongolian oak litter were collected from a farmland (44°0′16″ N,125°24′33″ E) and a secondary forest (43°47′6″ N,125°28′58″ E), respectively, in Changchun city, Jilin province of China. Yeast was used as food granulated over the substratum. Microcosm experiments were established using 180 mm × 120 mm plastic vessels to feed *Desoria ruseki* with the three food resources, each with five replicates, resulting in a total of 15 microcosms. Each microcosm was inoculated with 150 Collembola individuals and kept at 5°C with a diurnal light cycle (12 h light: 12 h dark), supplied with sufficient food and sterile water once a week. After 30 days, eight adult individuals were randomly selected from each microcosm for microbial DNA analyses, and an additional 20 adults were selected for bulk stable isotope analysis. In addition, the food substrates were collected from each microcosm, to investigate microbial communities and stable isotopes of the food. Collembola and food samples were stored at −80°C prior to DNA extraction and stable isotope analysis.

### Carbon and nitrogen stable isotope analysis

Collembola were washed three times with deionized water. Food substrates and Collembola were dried at 50°C for 48 h to constant weight before analysis. The stable isotope signatures of ^13^C and ^15^N of Collembola and food substrates were analyzed using a MAT253 stable isotope mass spectrometer (Thermo Fisher Scientific company) at the Analysis Test Center of Northeast Institute of Geography and Agroecology, Chinese Academy of Sciences. The precision of the ^13^C and ^15^N measurements was 0.15‰. Natural abundances of ^13^C and ^15^N isotopes were expressed by δ X notation. The standard substance was VPDB (Vienna Pee Dee Belemnite) for ^13^C and N_2_ for ^15^N as described previously (Hao et al., 2020).

### DNA extraction

Collembola were washed using 1.5% sodium hypochlorite for 15 s and subsequently rinsed for 1 min with sterilized water to remove surface microbial DNA contamination (Anslan et al., 2016). Total DNA was extracted using the Fast DNA® Spin Kit for Soil (MP Biomedicals, USA) following the protocol of the manufacturer from pools of eight individuals, to reduce individual variation. Microbial DNA of the food resources was extracted from 0.5 g of food materials from each microcosm, using the same DNA extraction procedure for the Collembola. Concentration of the extracted DNA was verified using a NanoDrop 2000 UV–vis spectrophotometer (Thermo Scientific, Wilmington, USA), and the quality of DNA was checked by 1% agarose gel electrophoresis. All operations were conducted under sterile conditions.

### 16S rRNA gene amplicon sequencing and bioinformatic analysis

DNA sequencing was performed by Majorbio Bio-Pharm Technology Co., Ltd. (Shanghai, China). The V3–V4 hypervariable region of the bacterial 16S rRNA gene was amplified using the specific barcoded primers 338F (5′-ACTCCTACGGGAGGCAGCAG-3′) and 806R (5′-GGACTACHVGGGTWTCTAAT-3′). Amplicons were generated through PCR reactions in triplicate, each in a 20 μl mixture containing 10 ng of template DNA, 0.8 μl of each primer (5 μM), 4 μl of 5 × FastPfu Buffer, 2 μl of 2.5 mM dNTPs, and 0.4 μl of FastPfu Polymerase, to which ddH_2_O was added to make the volume up to 20 μl. Thermocycler PCR system (GeneAmp®9,700, ABI, USA) settings were as following: initial denaturation for 5 min at 95°C, followed by 35 amplification cycles of 95°C for 30 s, 58°C for 30 s, 72°C for 30 s, with a final extension at 72°C for 5 min (Zhu et al., 2018). The PCR products were checked with a 2% agarose gel *via* electrophoresis. Positive PCR products were further purified using the AxyPrep DNA Gel Extraction Kit (Axygen Biosciences, Union City, CA, USA) and quantified using QuantiFluor™-ST (Promega, USA). Purified amplicons were pooled in equimolar and paired-end sequenced (2 × 300 bp) on Illumina MiSeq platform (Illumina, San Diego, USA).

The paired-end raw reads were demultiplexed, quality-filtered by Fastp, and merged by Flash. Qiime2 (v.2019.10) was used to process and analyze the sequences.^1^ Chimeric sequence identification and Amplicon Sequencing Variant (ASV) clustering were performed using the DADA2 pipeline (Callahan et al., 2016). The ASV taxonomy was assigned using the SILVA 16S rRNA gene database (v.138; Quast et al., 2013) with a confidence threshold of 70% using the RDP classifier. After removing mitochondria and chloroplast sequences, the resulting ASV table was rarefied to 19,083 sequences per sample for even sequencing depths among samples and used in downstream community analyses.

### Statistical analysis

Alpha diversity was estimated using Chao 1 richness index and Shannon index (Hugerth and Andersson, 2017). Kruskal–Wallis tests followed by Dunn test were performed to test for differences in bacterial alpha diversity. Box plot of bacterial alpha diversity and stacked bargraphs of community composition were drawn using the “ggplot2” package in R environment (v. 4.0.3). The ASVs present in at least 80% of the samples within each treatment was defined as the core microbiota of Collembola. The ASVs present in at least one read in one sample in each of Collembola and food treatments were defined as their shared ASVs, which were visualized using Venn diagram by Venny (v.2.1^2^). Differences in relative abundance of bacterial families between Collembola and food were analyzed with STAMP software (Parks et al., 2014). Linear discriminant analysis (LDA) with a threshold logarithmic LDA score of 2.0 was performed to identify significant differences of bacterial genera among treatments, and Kruskal–Wallis tests were performed to test for differences in bacterial relative abundances. Differences between bacterial communities were visualized using principal coordinate analysis (PCoA) based on Weighted-Unifrac metrics, and the PERMANOVA was performed to test whether bacterial communities differed between treatments (*p* < 0.05) using the R package “vegan.” To identify the most important bacterial taxa that likely contribute to the stable isotope enrichment in Collembola, random forest regression was used with the R package “randomForest.” The importance of bacterial taxa was determined by assessing the increase in the mean square error (MSE) and Nodepurity. Pearson’s correlation analysis was conducted to explore relationships between stable isotope enrichment in Collembola and their associated bacterial genera and diversity. The δ^13^C and δ^15^N values of Collembola were normalized by the food to determine the turnover and enrichment of their stable isotope. The differences in isotope enrichment in Collembola provided by different food resources were analyzed using one-way ANOVA, followed by Tukey honestly significant difference (HSD) test.

## Results

### Microbial diversity of cold-adapted Collembola

**Figure 2:**
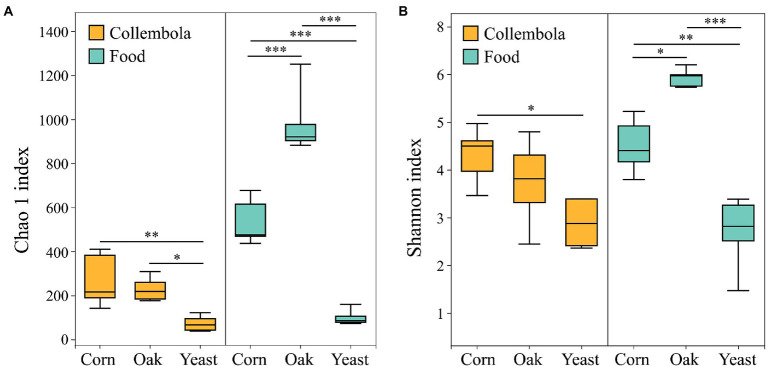
Box plot of the bacterial diversity of cold-adapted Collembola and food estimated by **(A)** Chao 1 richness index and **(B)** Shannon index. *** denotes *p* < 0.001; ** denotes *p* < 0.01; * denotes *p* < 0.05 based on Kruskal–Wallis test.

**Figure 3:**
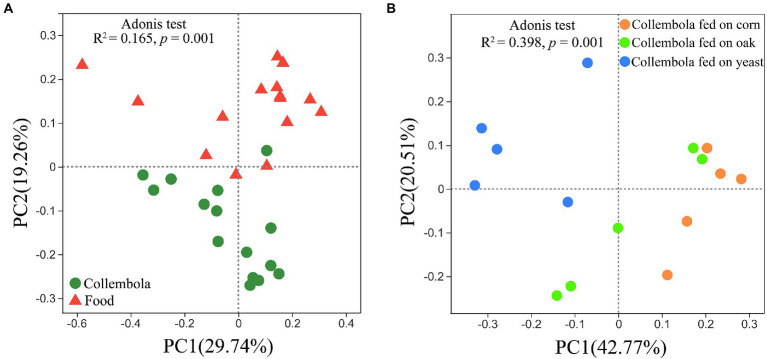
Bacterial community structure in cold-adapted Collembola and food types. Weighted-UniFrac principal coordinate analysis (PCoA) plots describe the differences in bacterial community structure between Collembola and food samples **(A)**, as well as the differences in bacterial community between Collembola fed with different food resources **(B)**.

A total of 796,292 and 899,341 high quality sequences were obtained from cold-adapted Collembola (*n* = 15) and food (*n* = 15; Figure 1) samples, respectively. A rarefaction curve analysis indicated sufficient coverage for the microbial sequences of Collembola and food samples (Supplementary Figure S1). A total of 1,557 and 3,969 ASVs were obtained across Collembola and food samples, respectively. Collembola fed on corn litter exhibited the highest microbial diversity as measured by Chao 1 estimated richness and the Shannon index (Kruskal–Wallis test, *p* < 0.05). In contrast, the Collembola fed on yeast had the lowest Chao1 richness and Shannon index values of all tested groups (Kruskal–Wallis test, *p* < 0.05; Figures 2A,B). The alpha diversity in bacterial species richness of cold-adapted Collembola showed a pronounced reduction compared to food as demonstrated by Chao 1 index (Kruskal–Wallis test, *p* < 0.05; Figure 2A). In addition, PCoA based on Weighted-Unifrac metrics indicated that the different food resources resulted in significant differences of bacterial communities in Collembola (Adonis test, *R*^2^ = 0.398, *p* < 0.01; Figure 3B).

### Microbial community composition of cold-adapted Collembola

**Figure 4:**
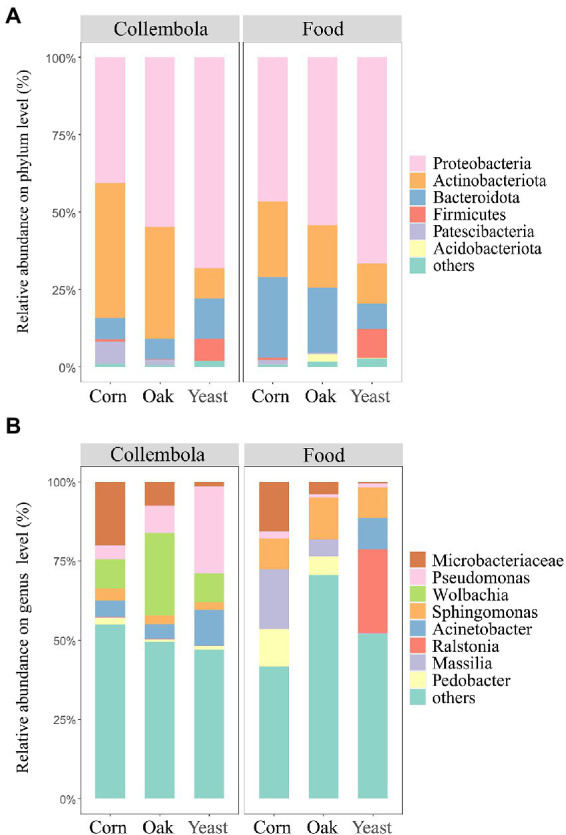
Bacterial community composition in cold-adapted Collembola and food. Panel **(A)** shows the bacterial community composition of Collembola at phylum level. Bacterial phyla with relative abundance <1% were categorized into ‘others’. Panel **(B)** shows the bacterial community composition of Collembola at the genus level. Bacterial genera with relative abundance <10% were categorized into ‘others’.

The most abundant bacterial phyla associated with Collembola were *Proteobacteria* (55.0% of total reads), *Actinobacteriota* (29.2%) and *Bacteroidota* (8.9%), comprising 93.1% of the bacterial ASVs associated with Collembola (Figure 4A). At the genus level, the bacterial community was predominated by *Wolbachia* (14.8% of total reads), *Pseudomonas* (13.5%), a representative of *Microbacteriaceae* (9.6%), and *Acinetobacter* (7.0%; Figure 4B). A core microbiota of 6 ASVs was shared in all Collembola treatments, accounting for 9.6% of the total reads.

Food resources had large impacts on bacterial community compositions associated with the cold-adapted Collembola. Bacterial communities associated with Collembola fed on corn litter and mongolian oak litter were predominantly colonized by the phylum *Proteobacteria* (40.6 and 54.8%, respectively) and *Actinobacteriota* (43.7 and 36.1%, respectively). Corn litter and mongolian oak litter feeders harbored a larger proportion of *Actinobacteriota*, compared to yeast feeders (Kruskal–Wallis test, *p* < 0.05). Mongolian oak litter feeders had a higher relative abundance of *Proteobacteria* (54.4%), compared to corn litter feeders (46.6%). Yeast feeders predominantly comprised *Proteobacteria* (68.2%) and *Bacteroidota* (12.9%), harboring the highest relative abundance of *Proteobacteria* and *Firmicutes* and the lowest relative abundance of *Patescibacteria* (0.001%), compared to the other feeders (Kruskal–Wallis test, *p* < 0.05). Additionally, LDA revealed that 32 bacterial genera of cold-adapted Collembola significantly differed among the three food resource treatments (Supplementary Figure S2). Corn litter feeders were predominated by a representative of *Microbacteriaceae* (20.0%) and had the highest relative abundance of this population compared to the other feeders (Kruskal–Wallis test, *p* < 0.05). Mongolian oak litter feeders were predominated by *Wolbachia* (26.1%) and had the highest relative abundance of *Wolbachia*, compared to the other groups (Figure 4B). Corn litter and mongolian oak litter feeders harbored a larger proportion of *Nocardioides* (5.3 and 4.2%, respectively) and *Aeromicrobium* (2.6 and 3.6%, respectively), compared to the yeast feeders (Kruskal–Wallis test, *p* < 0.05). Yeast feeders were predominated by *Pseudomonas* (27.5%) and *Acinetobacter* (11.2%), whereas the relative abundance of *Pseudomonas* in yeast feeders was significantly higher than that in the other feeders (Kruskal–Wallis test, *p* < 0.05).

### Differences of microbiota in cold-adapted Collembola and food

PCoA based on the Weighted-Unifrac revealed a significant separation of bacterial communities of Collembola from those in the food resources (Adonis test, R^2^ = 0.165, *p* < 0.01; Figure 3A). The relative abundance of two predominant phyla, *Bacteroidota* and *Acidobacteriota*, was significantly lower in food compared to Collembola (Kruskal–Wallis test, *p* < 0.05). Differences in bacterial families between Collembola and food were described in Supplementary Figure S3. The relative abundances of *Pseudomonadaceae*, *Anaplasmataceae*, *Moraxellaceae*, *Comamonadaceae*, and *Enterobacteriaceae* in Collembola were significantly higher than those in their food (Kruskal–Wallis test, *p* < 0.05; Supplementary Figure S3). However, a large number of ASVs in cold-adapted Collembola were shared with their food, and the shared ASV number in corn litter and mongolian oak litter feeders was larger than that of the yeast feeders (Supplementary Figure S4).

### Stable isotopic enrichment of cold-adapted Collembola

**Figure 5:**
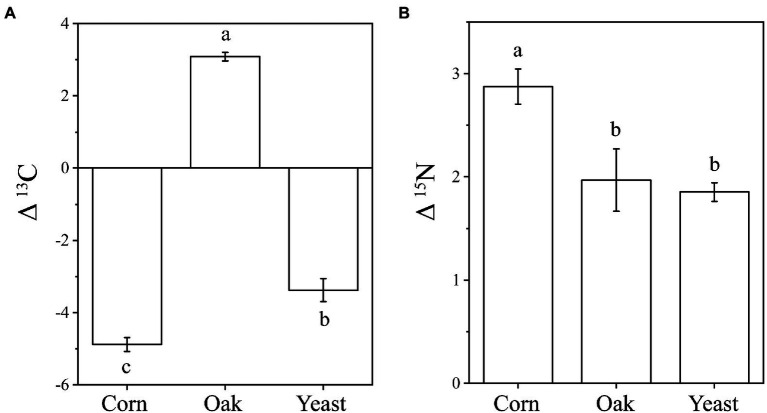
Stable isotopic enrichment of δ^13^C **(A)** and δ^15^N **(B)** in cold-adapted Collembola fed with different food resources. Different letters indicate significant differences between treatments (*p* < 0.05) according to Tukey HSD test.

**Figure 6:**
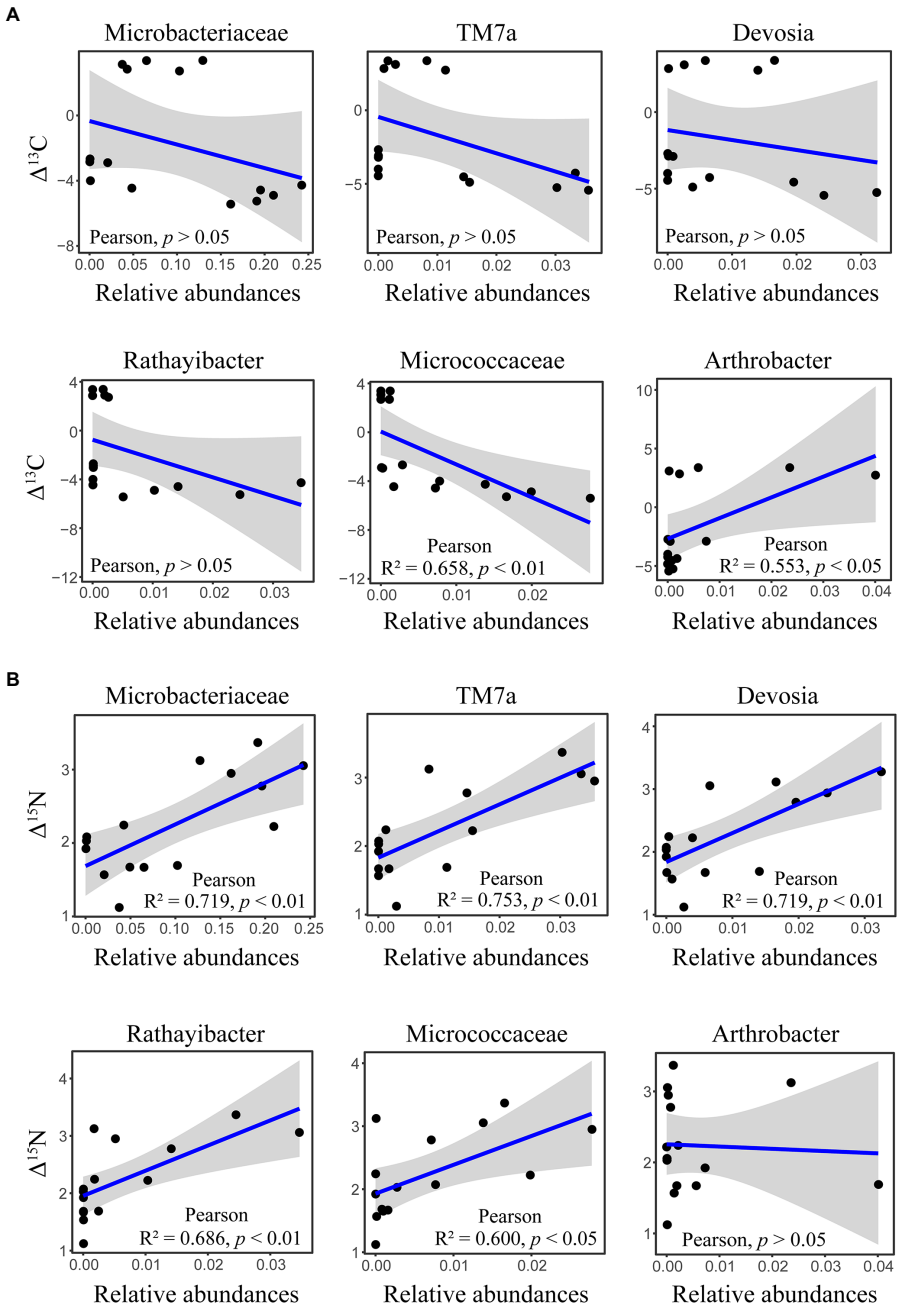
Correlation between important microbial genera and stable isotopic enrichment in cold-adapted Collembola. Panel **(A)** shows correlation between the relative abundance of *Microbacteriaceae*, TM7a, *Devosia*, *Rathayibacter*, *Micrococcaceae*, and *Arthrobacter*, and δ^13^C enrichment in Collembola. Panel **(B)** shows correlation between relative abundance of *Microbacteriaceae*, TM7a, *Devosia*, *Rathayibacter*, *Micrococcaceae*, and *Arthrobacter*, and δ^15^N enrichment in Collembola.

The δ^13^C and δ^15^N signatures of cold-adapted Collembola and food were described in Supplementary Table S1. Cold-adapted Collembola enriched −4.9, 3.1, and − 3.4 δ units for δ^13^C and 2.9, 2.0, and 1.9 δ units for δ^15^N after feeding on corn litter, mongolian oak litter, and yeast, respectively (Figures 5A,B). Collembola fed on mongolian oak litter had the highest δ^13^C enrichment, while the δ^15^N enrichment of corn litter feeders was significantly higher than that of the other feeders (one-way ANOVA, *p* < 0.05; Figures 5A,B). Interestingly, the δ^15^N enrichment in cold-adapted Collembola positively correlated with Shannon diversity index of microbiota associated with Collembola (Pearson, R^2^ = 0.403, *p* < 0.05). The genus *Arthrobacter* was positively correlated with the δ^13^C enrichment in Collembola. However, an uncharacterized representative of *Micrococcaceae* negatively correlated with the δ^13^C enrichment in Collembola (Figure 6A; Supplementary Figure S5). An uncharacterized representative of *Microbacteriaceae*, TM7a, *Devosia*, *Rathayibacter*, and an uncharacterized representative of *Micrococcaceae* were the most important bacterial populations that positively correlated with the δ^15^N enrichment in Collembola (Figure 6B; Supplementary Figure S6). The correlation between other microbial genera and stable isotopic enrichment of cold-adapted Collembola was described in Supplementary Table S2.

## Discussion

### Effect of different food resources on microbiota of cold-adapted Collembola

Diet plays an important role in shaping the microbiota of soil animals. It has previously been reported that soil invertebrates such as Collembola and earthworms develop a different gut microbiota when reared on different foods (Knapp et al., 2009; Xiang et al., 2019a). However, the effect of food nature on microbiota of the soil animals active in limited food and low temperatures winter is not well-studied. Our study demonstrates that variation in food resource supply influences the microbial community associated with cold-adapted Collembola, *Desoria ruseki*. Gut microbial diversity is critical in maintaining stability of intestinal microbiome and functioning of the host (Brune, 2014; Łukasik et al., 2017). Cold-adapted Collembola fed on corn litter and mongolian oak litter had more diverse bacteria, compared to the Collembola fed on yeasts (Figure 2), suggesting that microbiota of Collembola fed on the two litters has increased functional diversity (Engel and Moran, 2013). In contrast to the litters of corn and mongolian oak comprised of recalcitrant lignocellulosic material, the yeast diet is relatively simple, and this may explain the high bacterial diversity in litter feeders because that complex food resource contributes to diverse microbiota of soil arthropods (Xiang et al., 2019a). Corn litter as the agriculture grain residuals, however, leads to the highest bacterial diversity in Collembola; this can promote the stability of the microbiota of Collembola and may contribute to an important role in food digestion and nutrient absorption of Collembola fed on corn litter (Engel and Moran, 2013; Bahrndorff et al., 2018). In addition, the dissimilar microbial community structure of cold-adapted Collembola in response to different foods (Figure 3B) is consistent with the results of previous studies on earthworms (Liu et al., 2017). This suggests that different foods alter the microbial community diversity associated with soil animal active in winter, which could further affect the growth and health of the host.

The microbiota of cold-adapted Collembola predominantly comprised *Proteobacteria*, *Actinobacteriota*, and *Bacteroidota* (Figure 4A), which is consistent with the findings of previous studies on other Collembola species (Zhu et al., 2018; Ding et al., 2019). Collembola fed on corn litter and mongolian oak litter harbored a larger proportion of an uncharacterized genus affiliated to the *Microbacteriaceae*, compared to the yeast feeders. The family *Microbacteriaceae* is reported to be an important cellulolytic microbe in arthropod guts (Huang et al., 2012; Bashir et al., 2013), thus the large proportion of *Microbacteriaceae* in litter feeding Collembola may help in decomposition of cellulosic substrate. The relative abundance of *Wolbachia* was higher in Collembola fed on mongolian oak litter, compared to Collembola fed on corn litter and yeast. *Wolbachia* is an important endosymbiont commonly observed in the microbiota of arthropods and plays an essential role in regulating the reproductive behavior of the host (Czarnetzki and Tebbe, 2004; Werren et al., 2008). The C:N ratio of food greatly affects the reproduction of Collembola (Larsen et al., 2008); Previous studies reported that mongolian oak litter has a high C:N ratio, compared to the corn litter (Chen et al., 2003; Chae et al., 2016). The mongolian oak litter with high C:N ratio was found to result in a high proportion of *Wolbachia* in cold-adapted Collembola, and it could therefore be hypothesized that a poor food source can make Collembola more susceptible to the infection, thus relative abundance of *Wolbachia* will increase. Yeast is usually applied as a common food resource for Collembola culture in laboratories. We found that *Firmicutes* were significantly enriched in cold-adapted Collembola fed on yeast, which is in line with the findings of Xiang et al. (2019a). In addition, a large proportion of *Pseudomonas* was found in the microbiota of the yeast feeders. *Pseudomonas* could participate in chitin digestion by producing chitinase, thereby benefiting the animal consumer retrieving nutrients from the yeast cells (Rosenau and Jaeger, 2000; Smrž and Čatská, 2010; Gong et al., 2018). These results suggest that food nature determines microbial composition of cold-adapted Collembola, and different food sources largely influence bacterial enrichment in cold-adapted Collembola. Collembola species have a core microbiota in the warming season (Ding et al., 2019), we also identified a core bacterial community comprising 6 ASVs inhabiting the cold-adapted Collembola species. Cold-adapted Collembola harbor a lower microbial richness and a smaller core microbiota, the core microbiota might be prevalent in arthropods active in cold environments and likely help animals in cold adaption strategy (Leo et al., 2021). Further studies are needed to compare the differences in core microbiota of Collembola living in cold winter and growing season.

The microbial community in soil animals is closely related to the microbial community of surrounding environments. Previous studies reported that Collembola share a large number of bacteria with the soil where they live (Ding et al., 2019; Bi et al., 2021). The microbiota of the surrounding environment greatly shapes the microbiota of soil fauna. A higher bacterial richness was identified in corn litter and mongolian oak litter, compared to the yeasts. The cold-adapted Collembola fed on the two litters had more shared bacterial populations with the microbiota of their foods (Supplementary Figure S4). This suggests that the microbiota of food could influence the microbiota of cold-adapted Collembola. Different from the findings of previous study that *Folsomia candida* share a small amount of bacterial populations with their foods (Xiang et al., 2019a), we found that cold-adapted Collembola, *Desoria ruseki* had a large number of bacteria in common with their foods. It is reported that winter-active Collembola feed on certain bacteria from environments (Hao et al., 2020), we speculate that the microorganisms of environments could be important foods for Collembola living at low temperature in winter with limited food. The chemical composition or the microorganisms associated with a food source leads to modulation of microbial community structures of soil animals, as has also been demonstrated in previous reports (Knapp et al., 2009; Xiang et al., 2019a). However, which factor, the chemical composition or the microorganisms associated with a food source, is more important in shaping the microbial community of Collembola remains unknown. Further study should be directed toward uncovering the effect of these factors in shaping the microbiota of cold-adapted Collembola.

### Comparison between cold-adapted Collembola and food microbiota

The microbial community of cold-adapted Collembola was found to be noticeably different from that of their foods (Figure 3A), and similar results have been found in other Collembola (Zhu et al., 2018; Ding et al., 2019). Deterministic processes dominantly drive the microbiota in soil animals such as nematodes, oribatid mites, Collembola, and earthworms (Berg et al., 2016; Gong et al., 2018; Ding et al., 2019; Zhu et al., 2021; Gong et al., 2022). Specific conditions, particularly the conditions in the gut of soil animals have a large effect on microbiota, due to pH and oxygen availability in the gut, resulting in selection for certain bacteria from their foods or environments (Engel and Moran, 2013). The relative abundance of the families *Pseudomonadaceae*, *Moraxellaceae*, *Comamonadaceae*, and *Enterobacteriaceae* was significantly increased in cold-adapted Collembola, compared to their foods (Supplementary Figure S3), likely because the specialized gut microhabitat exerts a strong selective pressure on these microbes. Most of these families have been found to be associated with development and health of arthropods and benefit the host (Smrž and Čatská, 2010). For example, the *Enterobacteriaceae* is an important gut microbiota population that is relevant to litter decomposition as contributed by nematodes, Collembola, beetles, and earthworms (Wüst et al., 2011; Dirksen et al., 2016; Ding et al., 2019; Kudo et al., 2019).

### Relationship between microbiota and isotopic enrichment in cold-adapted Collembola

Few studies have investigated stable isotope of soil animals active in cold winter, especially in the case of Collembola (Hao et al., 2020). Measurements of δ^13^C can trace carbon sources used by consumers, as the values of δ^13^C are similar between consumer and food (Potapov et al., 2019). In contrast, the consumer is enriched in heavy nitrogen relative to food, and the values of δ^15^N increase from food to consumer by 3–4 δ units (Potapov et al., 2019). The present study showed that the higher δ^13^C and δ^15^N are in food, the higher δ^13^C and δ^15^N in cold-adapted Collembola (Supplementary Table S1), which is consistent with the results of previous studies on Collembola (Xiang et al., 2019a). Cold-adapted Collembola fed on mongolian oak litter had the highest enrichment of δ^13^C, compared to yeast and corn litter (Figure 5). However, Collembola fed on corn litter had the highest enrichment of δ^15^N, indicating that different food resources alter the stable isotopic enrichments in cold-adapted Collembola. Metabolic rate plays a key role in the stable isotopic turnover of the host (Zhu et al., 2018; Ek et al., 2019), and thus, the variation of stable isotopic enrichment could be explained by differences in metabolic rate in cold-adapted Collembola related to different foods. Animal metabolic pathways and the symbionts associated with animals contribute to nutrient turnover of the host (Cherel et al., 2005; Xiang et al., 2019a). Gut microbiota in soil animals can modulate metabolic rate by regulating host physiological functions such as food digestion and nutrition absorption (Xiang et al., 2019b; Ding et al., 2020). Thus, host-associated microbiota could be considered as important indicators for nutrient turnover and health of cold-adapted Collembola. Our findings suggest that the alterations in stable isotopic enrichment are associated with shifts in the microbiota of cold-adapted Collembola. The δ^15^N enrichment was positively correlated with the Shannon diversity index of Collembola-associated microbiota. Recently, a study has reported that soil Collembola harboring high gut microbial diversity have a high δ^15^N value (Xiang et al., 2019a), consistent with our results that cold-adapted Collembola had high bacterial diversity and a high δ^15^N enrichment. Gut microbiota can affect the assimilation of carbon and nitrogen isotopes in Collembola (Xiang et al., 2019a; Ding et al., 2020; Zhu et al., 2021), in turn altering their enrichment in the host. In our study, the genus *Arthrobacter* was positively correlated with the δ^13^C enrichment of cold-adapted Collembola (Figure 6A). The genus *Arthrobacter* is a beneficial microbe with its cellulolytic and lipolytic activities in arthropod guts (Briones-Roblero et al., 2017); therefore, *Arthrobacter* may be related to the utilization of carbon sources by cold-adapted Collembola. However, an uncharacterized representative of *Micrococcaceae* was negatively correlated with the δ^13^C enrichment of cold-adapted Collembola. *Micrococcaceae* is reported to be negatively correlated with the development of the multicolored Asian lady beetle (*Harmonia axyridis*; Huang et al., 2022), *Micrococcaceae* may also contribute to an adverse effect on the turnover of carbon sources in cold-adapted Collembola. The δ^15^N enrichment of cold-adapted Collembola was positively correlated with the uncharacterized representative of *Microbacteriaceae*, TM7a, *Devosia*, *Rathayibacter*, and the uncharacterized representative of *Micrococcaceae* (Figure 6B). It could be hypothesized that these microbes contribute to promoting δ^15^N turnover of cold-adapted Collembola by participating in food digestion and nutrient absorption, in line with previous reports (Cherel et al., 2005; Engel and Moran, 2013). For example, the family *Microbacteriaceae* is a probiotic microbe in animal guts that can convert multimers into monomers (Bashir et al., 2013; Ikeda-Ohtsubo et al., 2018). *Devosia* is a genus of Gram-negative bacteria that can degrade complex food sources (Li et al., 2018; Liu et al., 2021). *Devosia* in gut of black soldier fly larvae [*Hermetia illucens* (L.)] is dominant bacteria that digest lignin and hemicellulose of rice straw (Liu et al., 2021). In addition, the genus TM7a was involved in the process of nitrogen transformation in environments (Cheng et al., 2022); TM7a could participate in the turnover of nitrogen isotope in Collembola and promote nitrogen enrichment in the host. However, to have a mechanistic understanding of how the gut microbiota may contribute to stable isotopic enrichment and turnover in soil animal host, further lab manipulative experiments are required in the future, e.g., by excluding gut bacteria using antibiotic. Taken together, the results of the present study hint at potential roles of the microbiota associated with cold-adapted Collembola in regulating host fitness and health. Our results further imply that food resource change due to land-use managements and agricultural activities may alter the microbiota of cold-adapted Collembola with implications for their fitness.

## Conclusion

In the present study we explored the effect of food sources on the microbiota of cold-adapted Collembola. Changes in microbial community composition and diversity of cold-adapted Collembola was strongly shaped by food nature. Different food resources resulted in variations in the stable isotopic enrichment in cold-adapted Collembola. Our results also provide novel insight into the potential role of gut microbiota in regulating the stable isotopic turnover of cold-adapted soil invertebrates. These findings will improve our knowledge regarding the comprehensive host-microbiota interactions of soil animals active in cold regions. Further research is needed to investigate metabolic functions of important microbes related to different food sources and nutrient turnover in cold-adapted Collembola.

## Data availability statement

The data presented in the study are deposited in the BioProject repository, accession number PRJNA815480 (http://www.ncbi.nlm.nih.gov/bioproject/815480).

## Author contributions

DHW and CH contributed to the design of the study. CH and LCF collected the Collembola in field. CH performed the experiments and wrote the original manuscript. CH, JLN, and NdJ analyzed the data. JLN, NdJ, DZ, BZ, and T-WC reviewed and edited the manuscript. All authors contributed to the article and approved the submitted version.

## Funding

This present study was supported by the Strategic Priority Research Program of Chinese Academy of Sciences (No. XDA28020201), the National Natural Sciences Foundation of China (Nos. 41671259 and 42071059), the Program of Introducing Talents of Discipline to Universities (No. B16011), and the National Science & Technology Fundamental Resources Investigation Program of China (No. 2018FY100300). NdJ and JLN were supported by Aalborg University. T-WC was supported by the Czech Academy of Sciences with the MSM project for research and mobility of starting researchers (MSM200962001). CH was supported by the China Scholarship Council (202106620026).

## Conflict of interest

The authors declare that the research was conducted in the absence of any commercial or financial relationships that could be construed as a potential conflict of interest.

## Publisher’s note

All claims expressed in this article are solely those of the authors and do not necessarily represent those of their affiliated organizations, or those of the publisher, the editors and the reviewers. Any product that may be evaluated in this article, or claim that may be made by its manufacturer, is not guaranteed or endorsed by the publisher.

## Footnotes

^1^ https://qiime2.org

^2^ https://bioinfogp.cnb.csic.es/tools/venny/index.html

## References

[ref1] Anslan S. Bahram M. Tedersoo L. (2016). Temporal changes in fungal communities associated with guts and appendages of Collembola as based on culturing and high-throughput sequencing. Soil Biol. Biochem. 96, 152–159. doi: 10.1016/j.soilbio.2016.02.006

[ref2] Bahrndorff S. de Jonge N. Hansen J. K. Lauritzen J. M. S. Spanggaard L. H. Sørensen M. H.. (2018). Diversity and metabolic potential of the microbiota associated with a soil arthropod. Sci. Rep. 8:2491. doi: 10.1038/s41598-018-20967-0, PMID: 29410494 PMC5802828

[ref3] Bashir Z. Kondapalli V. K. Adlakha N. Sharma A. Bhatnagar R. K. Chandel G.. (2013). Diversity and functional significance of cellulolytic microbes living in termite, pill-bug and stem-borer guts. Sci. Rep. 3:2558. doi: 10.1038/srep02558, PMID: 23990056 PMC3757366

[ref4] Berg M. Stenuit B. Ho J. Wang A. Parke C. Knight M.. (2016). Assembly of the *Caenorhabditis elegans* gut microbiota from diverse soil microbial environments. ISME J. 10, 1998–2009. doi: 10.1038/ismej.2015.253, PMID: 26800234 PMC5029150

[ref5] Bi Q. F. Jin B. J. Zhu D. Jiang Y. G. Zheng B. X. O'Connor P.. (2021). How can fertilization regimes and durations shape earthworm gut microbiota in a long-term field experiment? Ecotox. Environ. Saf. 224:112643. doi: 10.1016/j.ecoenv.2021.112643, PMID: 34411817

[ref6] Bokhorst S. Wardle D. A. (2014). Snow fungi as a food source for micro-arthropods. Eur. J. Soil Biol. 60, 77–80. doi: 10.1016/j.ejsobi.2013.11.006

[ref7] Briones-Roblero C. I. Rodríguez-Díaz R. Santiago-Cruz J. A. Zúñiga G. Rivera-Orduña F. N. (2017). Degradation capacities of bacteria and yeasts isolated from the gut of *Dendroctonus rhizophagus* (Curculionidae: Scolytinae). Folia Microbiol. 62, 1–9. doi: 10.1007/s12223-016-0469-4, PMID: 27544667

[ref8] Brune A. (2014). Symbiotic digestion of lignocellulose in termite guts. Nat. Rev. Microbiol. 12, 168–180. doi: 10.1038/nrmicro3182, PMID: 24487819

[ref9] Buse T. Ruess L. Filser J. (2013). New trophic biomarkers for Collembola reared on algal diets. Pedobiologia 56, 153–159. doi: 10.1016/j.pedobi.2013.03.005

[ref10] Callahan B. J. McMurdie P. J. Rosen M. J. Han A. W. Johnson A. J. A. Holmes S. P. (2016). DADA2: high-resolution sample inference from illumina amplicon data. Nat. Methods 13, 581–583. doi: 10.1038/NMETH.3869, PMID: 27214047 PMC4927377

[ref11] Chae H. M. Cha S. Lee S. H. Choi M. J. Shim J. K. (2016). Age-related decomposition of *Quercus mongolica* branches. Plant Ecol. 217, 945–957. doi: 10.1007/s11258-016-0620-y

[ref12] Chen X. Cabrera M. L. Zhang L. Shi Y. Shen S. M. (2003). Long-term decomposition of organic materials with different carbon/nitrogen ratios. Commun. Soil Sci. Plant Anal. 34, 41–54. doi: 10.1081/CSS-120017414

[ref13] Cheng C. He Q. Zhang J. Chai H. X. Yang Y. J. Pavlostathis S. G.. (2022). New insight into ammonium oxidation processes and mechanisms mediated by manganese oxide in constructed wetlands. Water Res. 215:118251. doi: 10.1016/j.watres.2022.118251, PMID: 35278914

[ref14] Cherel Y. Hobson K. A. Bailleul F. Groscolas R. (2005). Nutrition, physiology, and stable isotopes: new information from fasting and molting penguins. Ecology 86, 2881–2888. doi: 10.1890/05-0562

[ref15] Chevalier C. Stojanović O. Colin D. J. Suarez-Zamorano N. Tarallo V. Veyrat-Durebex C.. (2015). Gut microbiota orchestrates energy homeostasis during cold. Cells 163, 1360–1374. doi: 10.1016/j.cell.2015.11.004, PMID: 26638070

[ref16] Czarnetzki A. B. Tebbe C. C. (2004). Detection and phylogenetic analysis of *Wolbachia* in Collembola. Environ. Microbiol. 6, 35–44. doi: 10.1046/j.1462-2920.2003.00537.x, PMID: 14686939

[ref17] Ding J. Liu J. Chang X. B. Zhu D. Lassen S. B. (2020). Exposure of CuO nanoparticles and their metal counterpart leads to change in the gut microbiota and resistome of collembolans. Chemosphere 258:127347. doi: 10.1016/j.chemosphere.2020.127347, PMID: 32535433

[ref18] Ding J. Zhu D. Chen Q. L. Zheng F. Wang H. T. Zhu Y. G. (2019). Effects of long-term fertilization on the associated microbiota of soil collembolan. Soil Biol. Biochem. 130, 141–149. doi: 10.1016/j.soilbio.2018.12.015

[ref19] Dirksen P. Marsh S. A. Braker I. Heitland N. Wagner S. Nakad R.. (2016). The native microbiome of the nematode *Caenorhabditis elegans*: gateway to a new host-microbiome model. BMC Biol. 14:38. doi: 10.1186/s12915-016-0258-1, PMID: 27160191 PMC4860760

[ref20] Ek C. Yu Z. Y. Garbaras A. Oskarsson H. Wiklund A. K. E. Kumblad L.. (2019). Increase in stable isotope ratios driven by metabolic alterations in amphipods exposed to the beta-blocker propranolol. PLoS One 14:e0211304. doi: 10.1371/journal.pone.0211304, PMID: 31095563 PMC6522046

[ref21] Engel P. Moran N. A. (2013). The gut microbiota of insects-diversity in structure and function. FEMS Microbiol. Rev. 37, 699–735. doi: 10.1111/1574-6976.12025 23692388

[ref22] Gong X. Chen T.-W. Zhang L. L. Pižl V. Tajovský K. Devetter M. (2022). Gut microbiome reflect adaptation of earthworms to cave and surface environments. Anim.Microbiome 4:47. doi: 10.1186/s42523-022-00200-0, PMID: 35932082 PMC9356433

[ref23] Gong X. Chen T.-W. Zieger S. L. Bluhm C. Heidemann K. Schaefer I.. (2018). Phylogenetic and trophic determinants of gut microbiota in soil oribatid mites. Soil Biol. Biochem. 123, 155–164. doi: 10.1016/j.soilbio.2018.05.011

[ref24] Hågvar S. (2010). A review of Fennoscandian arthropods living on and in snow. Eur. J. Entomol. 107, 281–298. doi: 10.14411/eje.2010.037

[ref25] Hao C. Chen T.-W. Wu Y. G. Chang L. Wu D. H. (2020). Snow microhabitats provide food resources for winter-active Collembola. Soil Biol. Biochem. 143:107731. doi: 10.1016/j.soilbio.2020.107731

[ref26] Hu J. Zhao H. T. Wang Y. Yin Z. F. Kang Y. J. (2020). The bacterial community structures in response to the gut passage of earthworm (*Eisenia fetida*) feeding on cow dung and domestic sludge: illumina high-throughput sequencing-based data analysis. Ecotox. Environ. Saf. 190:110149. doi: 10.1016/j.ecoenv.2019.110149, PMID: 31901807

[ref27] Huang S. W. Sheng P. Zhang H. Y. (2012). Isolation and identification of cellulolytic bacteria from the gut of *Holotrichia parallela* larvae (Coleoptera: Scarabaeidae). Int. J. Mol. Sci. 13, 2563–2577. doi: 10.3390/ijms13032563, PMID: 22489111 PMC3317674

[ref28] Huang Z. D. Zhu L. Lv J. Pu Z. X. Zhang L. P. Chen G. Q.. (2022). Dietary effects on biological parameters and gut microbiota of *Harmonia axyridis*. Front. Microbiol. 12:818787. doi: 10.3389/fmicb.2021.818787, PMID: 35154044 PMC8828657

[ref29] Hugerth L. W. Andersson A. F. (2017). Analysing microbial community composition through amplicon sequencing: from sampling to hypothesis testing. Front. Microbiol. 8:1561. doi: 10.3389/fmicb.2017.01561, PMID: 28928718 PMC5591341

[ref30] Ikeda-Ohtsubo W. Brugman S. Warden C. H. Rebel J. M. J. Folkerts G. Pieterse C. M. J. (2018). How can we define “optimal microbiota?”: a comparative review of structure and functions of microbiota of animals, fish, and plants in agriculture. Front. Nutr. 5:90. doi: 10.3389/fnut.2018.00090, PMID: 30333981 PMC6176000

[ref31] Kennedy S. R. Tsau S. Gillespie R. Krehenwinkel H. (2020). Are you what you eat? A highly transient and prey-influenced gut microbiome in the grey house spider *Badumna longinqua*. Mol. Ecol. 29, 1001–1015. doi: 10.1111/mec.15370, PMID: 32011756

[ref32] Knapp B. A. Podmirseg S. M. Seeber J. Meyer E. Insam H. (2009). Diet-related composition of the gut microbiota of *Lumbricus rubellus* as revealed by a molecular fingerprinting technique and cloning. Soil Biol. Biochem. 41, 2299–2307. doi: 10.1016/j.soilbio.2009.08.011

[ref33] Kudo R. Masuya H. Endoh R. Kikuchi T. Ikeda H. (2019). Gut bacterial and fungal communities in ground-dwelling beetles are associated with host food habit and habitat. ISME J. 13, 676–685. doi: 10.1038/s41396-018-0298-3, PMID: 30333525 PMC6461832

[ref34] Larsen J. Johansen A. Larsen S. E. Heckmann L. H. Jakobsen I. Krogh P. H. (2008). Population performance of collembolans feeding on soil fungi from different ecological niches. Soil Biol. Biochem. 40, 360–369. doi: 10.1016/j.soilbio.2007.08.016

[ref35] Leo C. Nardi F. Cucini C. Frati F. Convey P. Weedon J. T.. (2021). Evidence for strong environmental control on bacterial microbiomes of Antarctic springtails. Sci. Rep. 11:2973. doi: 10.1038/s41598-021-82379-x, PMID: 33536493 PMC7858589

[ref36] Li X. Y. Guo Y. P. Zhao L. H. Fan Y. Ji C. Zhang J. Y.. (2018). Protective effects of *Devosia* sp. ANSB714 on growth performance, immunity function, antioxidant capacity and tissue residues in growing-finishing pigs fed with deoxynivalenol contaminated diets. Food Chem. Toxicol. 121, 246–251. doi: 10.1016/j.fct.2018.09.007, PMID: 30201387

[ref37] Liu D. F. Lian B. Wu C. H. Guo P. J. (2017). A comparative study of gut microbiota profiles of earthworms fed in three different substrates. Symbiosis 74, 21–29. doi: 10.1007/s13199-017-0491-6

[ref38] Liu C. C. Wang C. W. Yao H. Y. Chapman S. J. (2021). Pretreatment is an important method for increasing the conversion efficiency of rice straw by black soldier fly larvae based on the function of gut microorganisms. Sci. Total Environ. 762:144118. doi: 10.1016/j.scitotenv.2020.144118, PMID: 33360472

[ref39] Liu Z. J. Yang X. G. Hubbard K. G. Lin X. M. (2012). Maize potential yields and yield gaps in the changing climate of Northeast China. Glob. Change Biol. 18, 3441–3454. doi: 10.1111/j.1365-2486.2012.02774.x

[ref40] Łukasik P. Newton J. A. Sanders J. G. Hu Y. Moreau C. S. Kronauer D. J. C.. (2017). The structured diversity of specialized gut symbionts of the new world army ants. Mol. Ecol. 26, 3808–3825. doi: 10.1111/mec.14140, PMID: 28393425

[ref41] Mikaelyan A. Dietrich C. Köhler T. Poulsen M. Sillam-Dussès D. Brune A. (2015). Diet is the primary determinant of bacterial community structure in the guts of higher termites. Mol. Ecol. 24, 5284–5295. doi: 10.1111/mec.13376, PMID: 26348261

[ref42] Oliver T. H. Morecroft M. D. (2014). Interactions between climate change and land use change on biodiversity: attribution problems, risks, and opportunities. *Wiley Interdiscip*. Rev.-Clim. Chang. 5, 317–335. doi: 10.1002/wcc.271

[ref43] Parks D. H. Tyson G. W. Hugenholtz P. Beiko R. G. (2014). STAMP: statistical analysis of taxonomic and functional profiles. Bioinformatics 30, 3123–3124. doi: 10.1093/bioinformatics/btu494, PMID: 25061070 PMC4609014

[ref44] Potapov A. M. Pollierer M. M. Salmon S. Šustr V. Chen T.-W. (2021). Multidimensional trophic niche revealed by complementary approaches: gut content, digestive enzymes, fatty acids and stable isotopes in Collembola. J. Anim. Ecol. 90, 1919–1933. doi: 10.1111/1365-2656.13511, PMID: 33914342 PMC8453724

[ref45] Potapov A. M. Tiunov A. V. Scheu S. (2019). Uncovering trophic positions and food resources of soil animals using bulk natural stable isotope composition. Biol. Rev. 94, 37–59. doi: 10.1111/brv.12434, PMID: 29920907

[ref46] Quast C. Pruesse E. Yilmaz P. Gerken J. Schweer T. Yarza P.. (2013). The Silva ribosomal RNA gene database project: improved data processing and web-based tools. Nucleic Acids Res. 41, D590–D596. doi: 10.1093/nar/gks1219, PMID: 23193283 PMC3531112

[ref47] Rosenau F. Jaeger K. E. (2000). Bacterial lipases from *pseudomonas*: regulation of gene expression and mechanisms of secretion. Biochimie 82, 1023–1032. doi: 10.1016/S0300-9084(00)01182-2, PMID: 11099799

[ref48] Sadaka-Laulan N. Ponge J. F. (2000). Influence of holm oak leaf decomposition stage on the biology of *Onychiurus sinensis* Stach (Collembola: Onychiuridae). Eur. J. Soil Biol. 36, 97–105. doi: 10.1016/S1164-5563(00)01051-7

[ref49] Scheunemann N. Maraun M. Scheu S. Butenschoen O. (2015). The role of shoot residues vs. crop species for soil arthropod diversity and abundance of arable systems. Soil Biol. Biochem. 81, 81–88. doi: 10.1016/j.soilbio.2014.11.006

[ref50] Sepulveda J. Moeller A. H. (2020). The effects of temperature on animal gut microbiomes. Front. Microbiol. 11:384. doi: 10.3389/fmicb.2020.00384, PMID: 32210948 PMC7076155

[ref51] Smrž J. Čatská V. (2010). Mycophagous mites and their internal associated bacteria cooperate to digest chitin in soil. Symbiosis 52, 33–40. doi: 10.1007/s13199-010-0099-6

[ref52] Susanti W. I. Widyastuti R. Scheu S. Potapov A. (2021). Trophic niche differentiation and utilisation of food resources in Collembola is altered by rainforest conversion to plantation systems. PeerJ 9:e10971. doi: 10.7717/peerj.10971, PMID: 33717699 PMC7934680

[ref53] Valle B. Cucini C. Nardi F. Caccianiga M. Gobbi M. Musciano M. D.. (2021). *Desoria calderonis* sp. nov., a new species of alpine cryophilic springtail (Collembola: Isotomidae) from the Apennines (Italy), with phylogenetic and ecological considerations. Eur. J. Taxon. 787, 32–52. doi: 10.5852/ejt.2021.787.1599

[ref54] Werren J. H. Baldo L. Clark M. E. (2008). *Wolbachia*: master manipulators of invertebrate biology. Nat. Rev. Microbiol. 6, 741–751. doi: 10.1038/nrmicro1969, PMID: 18794912

[ref55] Wüst P. K. Horn M. A. Drake H. L. (2011). Clostridiaceae and Enterobacteriaceae as active fermenters in earthworm gut content. ISME J. 5, 92–106. doi: 10.1038/ismej.2010.99, PMID: 20613788 PMC3105676

[ref56] Xiang Q. Zhu D. Chen Q. L. Delgado-Baquerizo M. Su J. Q. Qiao M.. (2019a). Effects of diet on gut microbiota of soil collembolans. Sci. Total Environ. 676, 197–205. doi: 10.1016/j.scitotenv.2019.04.104, PMID: 31048151

[ref57] Xiang Q. Zhu D. Chen Q. L. O'Connor P. Yang X. R. Qiao M.. (2019b). Adsorbed sulfamethoxazole exacerbates the effects of polystyrene (~2 μm) on gut microbiota and the antibiotic resistome of a soil collembolan. Environ. Sci. Technol. 53, 12823–12834. doi: 10.1021/acs.est.9b04795, PMID: 31593455

[ref58] Zettel J. Zettel U. Suter C. Streich S. Egger B. (2002). Winter feeding behaviour of *Ceratophysella sigillata* (Collembola: Hypogastruridae) and the significance of eversible vesicles for resource utilisation. Pedobiologia 46, 404–413. doi: 10.1078/0031-4056-00148

[ref59] Zhang B. Chang L. Ni Z. Callaham M. A. Jr. Sun X. Wu D. H. (2014). Effects of land use changes on winter-active Collembola in Sanjiang plain of China. Appl. Soil Ecol. 83, 51–58. doi: 10.1016/j.apsoil.2014.03.008

[ref60] Zhang B. Chang L. Ni Z. Sun X. Wu D. H. (2017). Directional migration of three *Desoria* species (Collembola: Isotomidae) on the snow surface in late winter. Eur. J. Soil Biol. 81, 64–68. doi: 10.1016/j.ejsobi.2017.06.009

[ref61] Zhang X. L. Lv P. C. Xu C. Huang X. R. Rademacher T. (2021). Dryness decreases average growth rate and increases drought sensitivity of Mongolia oak trees in North China. Agric. For. Meteorol. 308-309:108611. doi: 10.1016/j.agrformet.2021.108611

[ref62] Zhu D. Chen Q. L. An X. L. Yang X. R. Christie P. Ke X.. (2018). Exposure of soil collembolans to microplastics perturbs their gut microbiota and alters their isotopic composition. Soil Biol. Biochem. 116, 302–310. doi: 10.1016/j.soilbio.2017.10.027

[ref63] Zhu D. Delgado-Baquerizo M. Ding J. Gillings M. R. Zhu Y. G. (2021). Trophic level drives the host microbiome of soil invertebrates at a continental scale. Microbiome 9:189. doi: 10.1186/s40168-021-01144-4, PMID: 34544484 PMC8454154

