# Supplementary material for: Food origin influences microbiota and stable isotope enrichment profiles of cold-adapted Collembola (Desoria ruseki)
Source: Front Microbiol. 2022 Nov 24;13:1030429. doi: 10.3389/fmicb.2022.1030429 (PMC9730247; doi:10.3389/fmicb.2022.1030429)
Supplement: Supplementary file 1 [file Data_Sheet_1.docx]

**Supplementary Material**


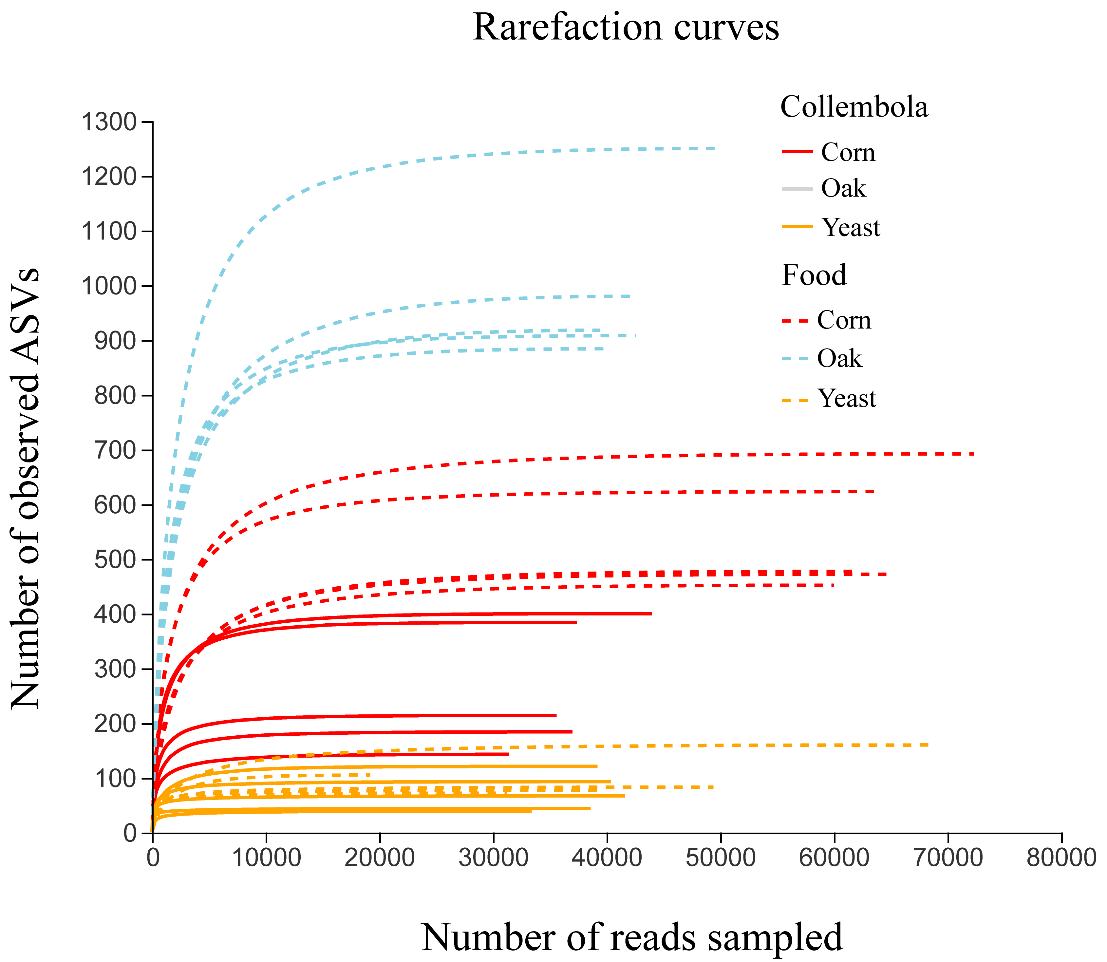


Supplementary Figure 1. The rarefaction curve based on the ASV level of microbial sequences of cold-adapted Collembola and food samples.


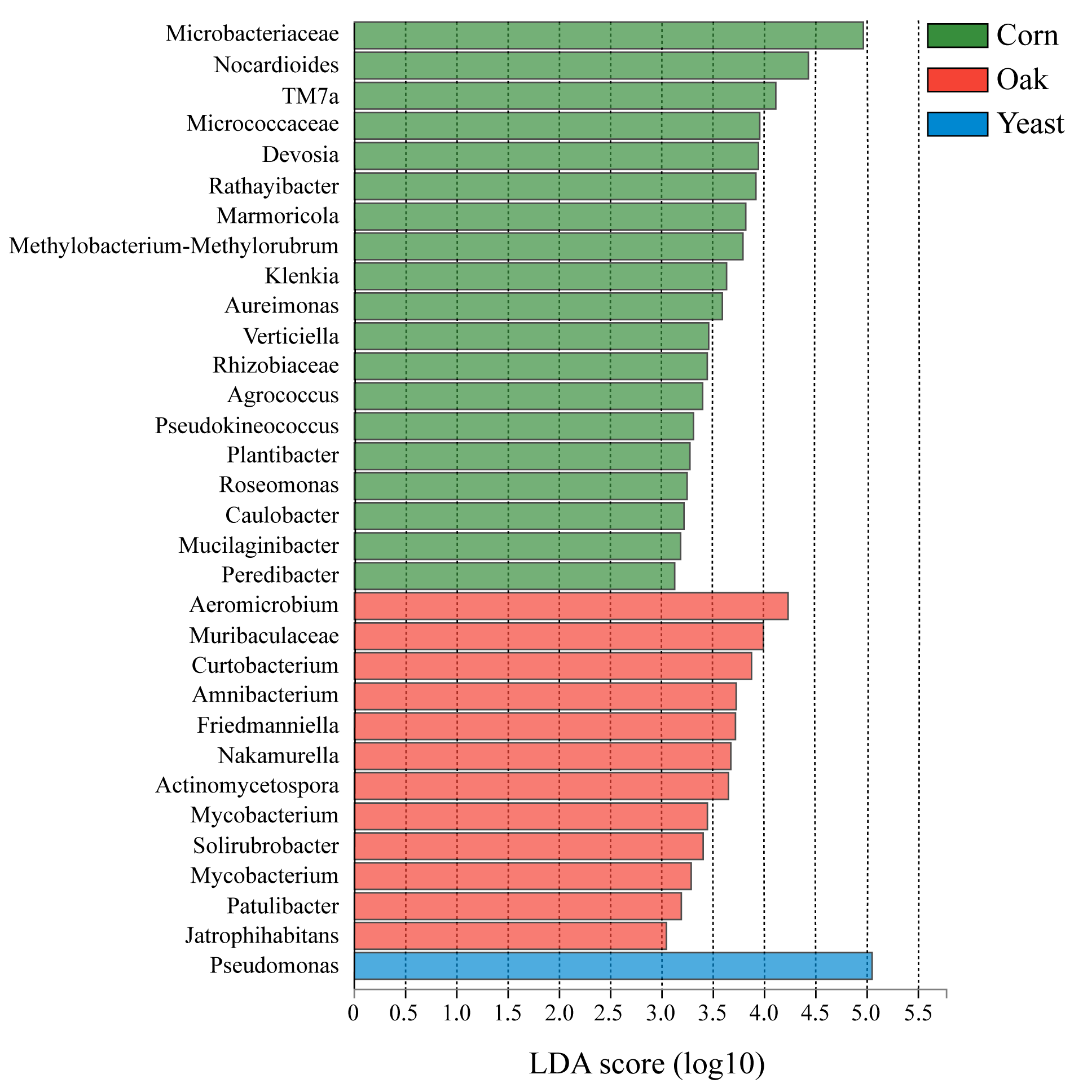


Supplementary Figure 2. Linear discriminant analysis (LDA) plot demonstrating the enrichment of bacterial genera of cold-adapted Collembola fed on three different food resources. The threshold value of the log LDA score for discriminating features was 2.0. LDA scores represent the degree of influence of significantly different taxa between different treatments.


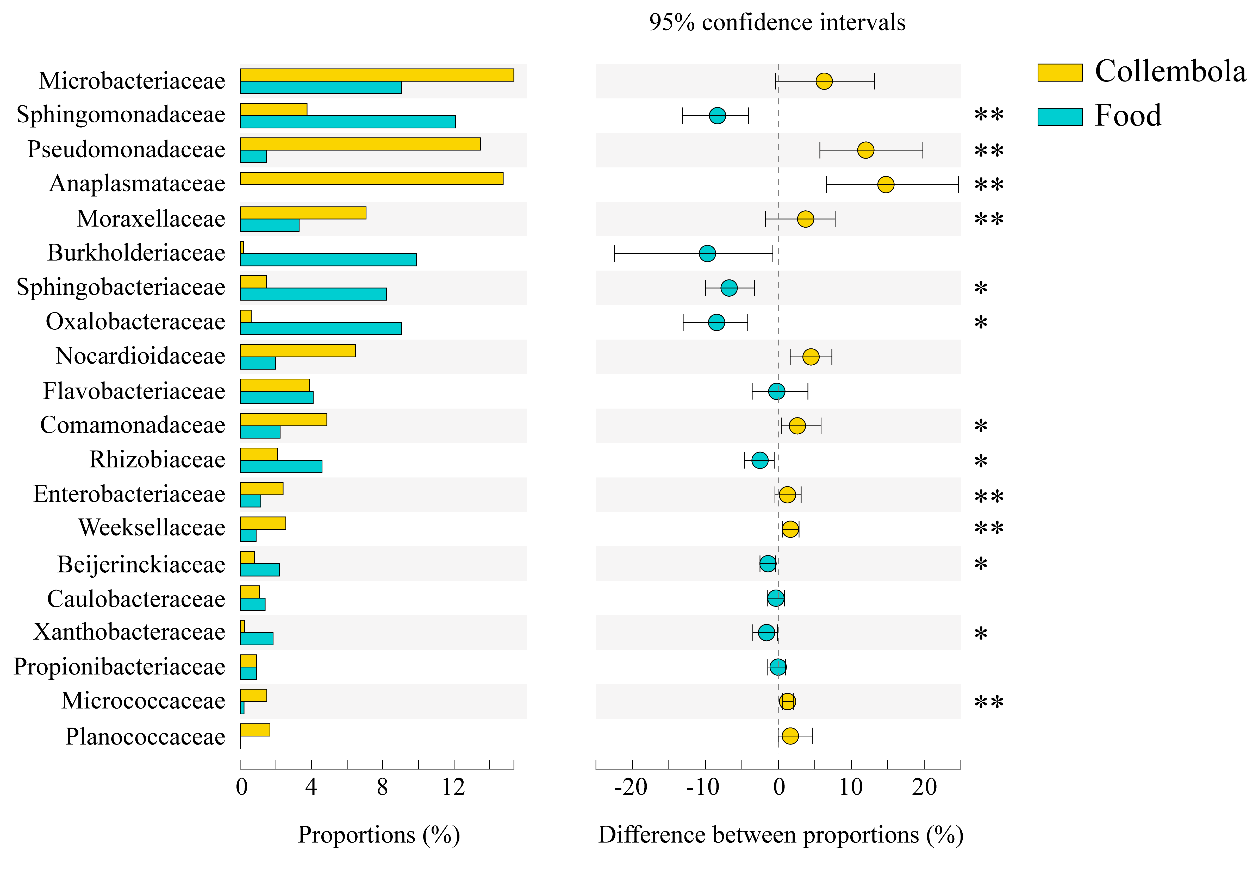


Supplementary Figure 3. A significant difference in bacterial families was observed between cold-adapted Collembola and foods. The most abundant 20 bacterial families in the Collembola and the food were presented. ** denotes *p* < 0.01; * denotes *p* < 0.05.


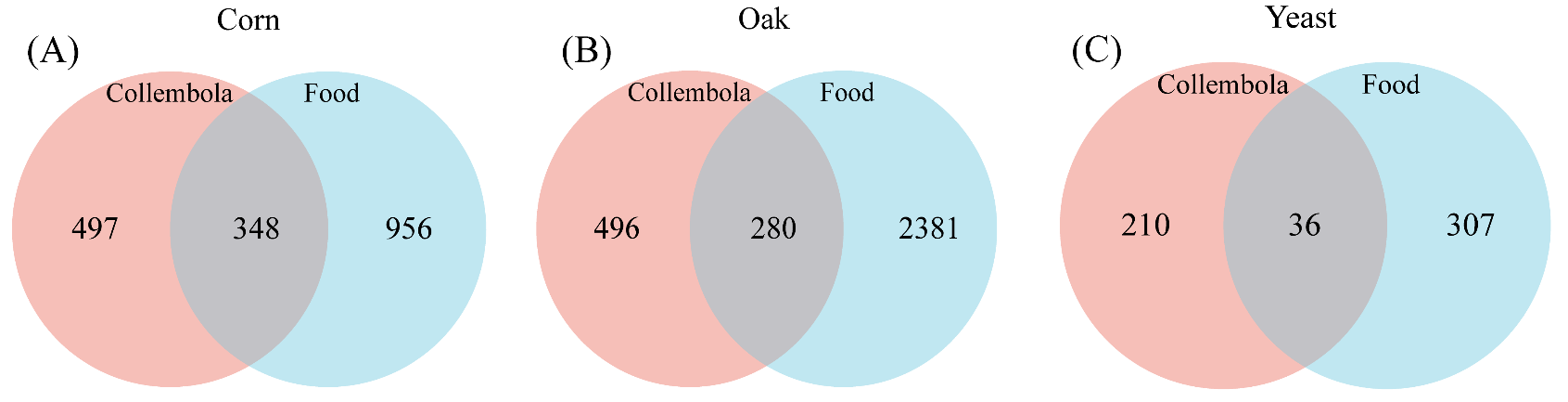


Supplementary Figure 4. Venn diagrams displaying the overlap of ASVs between different treatments. Panel (A), (B) and (C) shows ASVs overlap of between Collembola and food in corn litter, mongolian oak litter and yeast, respectively.


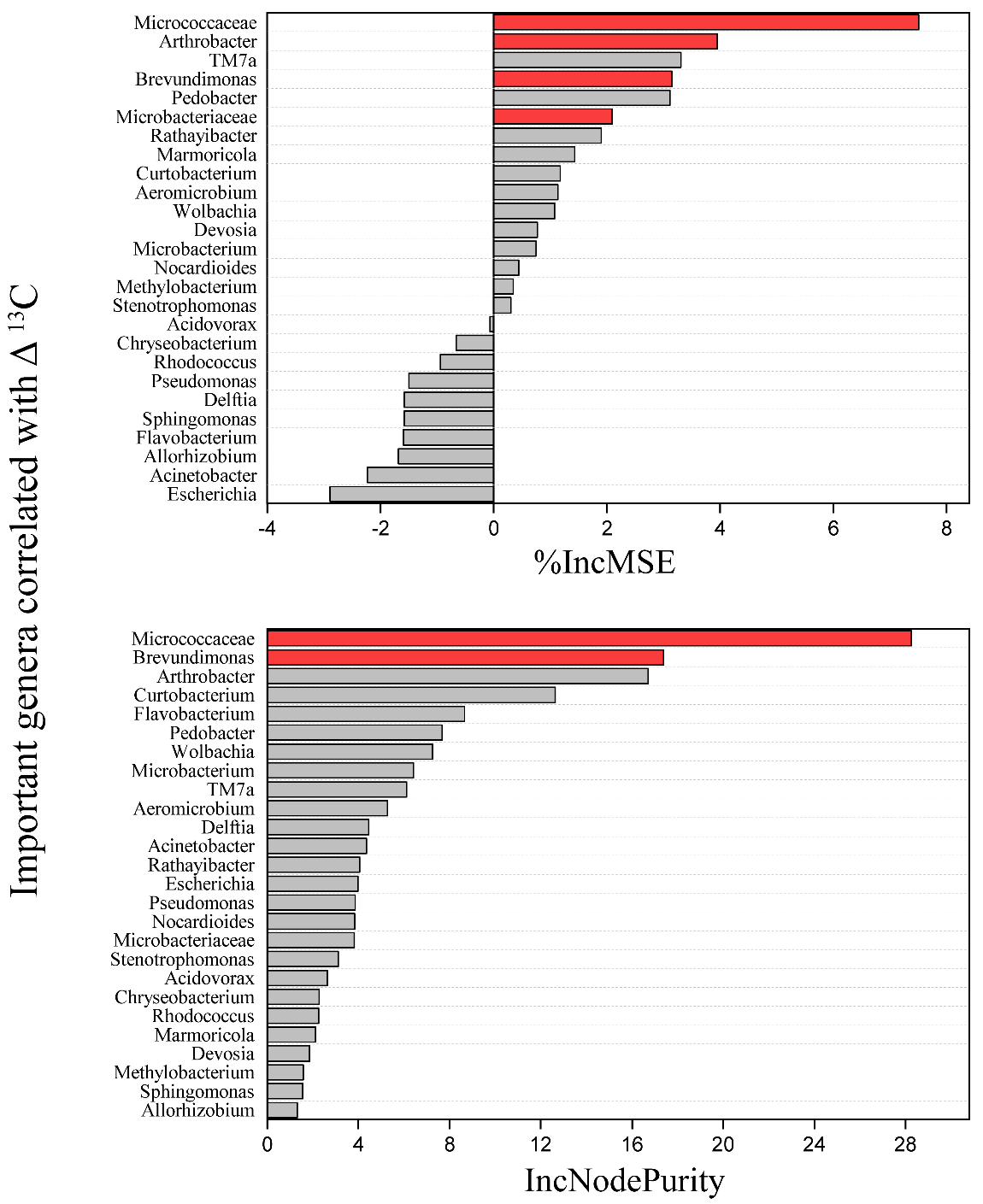


Supplementary Figure 5. Variable importance of the Random Forest regression model for the prediction of cold-adapted Collembola associated microbiota-Δ^13^C enrichment correlated with different food treatments. The variables show the bacterial genera with relative abundance > 1% in Collembola, ranked on the basis of their importance for the increase in the mean square error (MSE) and NodePurity. Genera with a red background indicate significantly important species (*p* < 0.05).


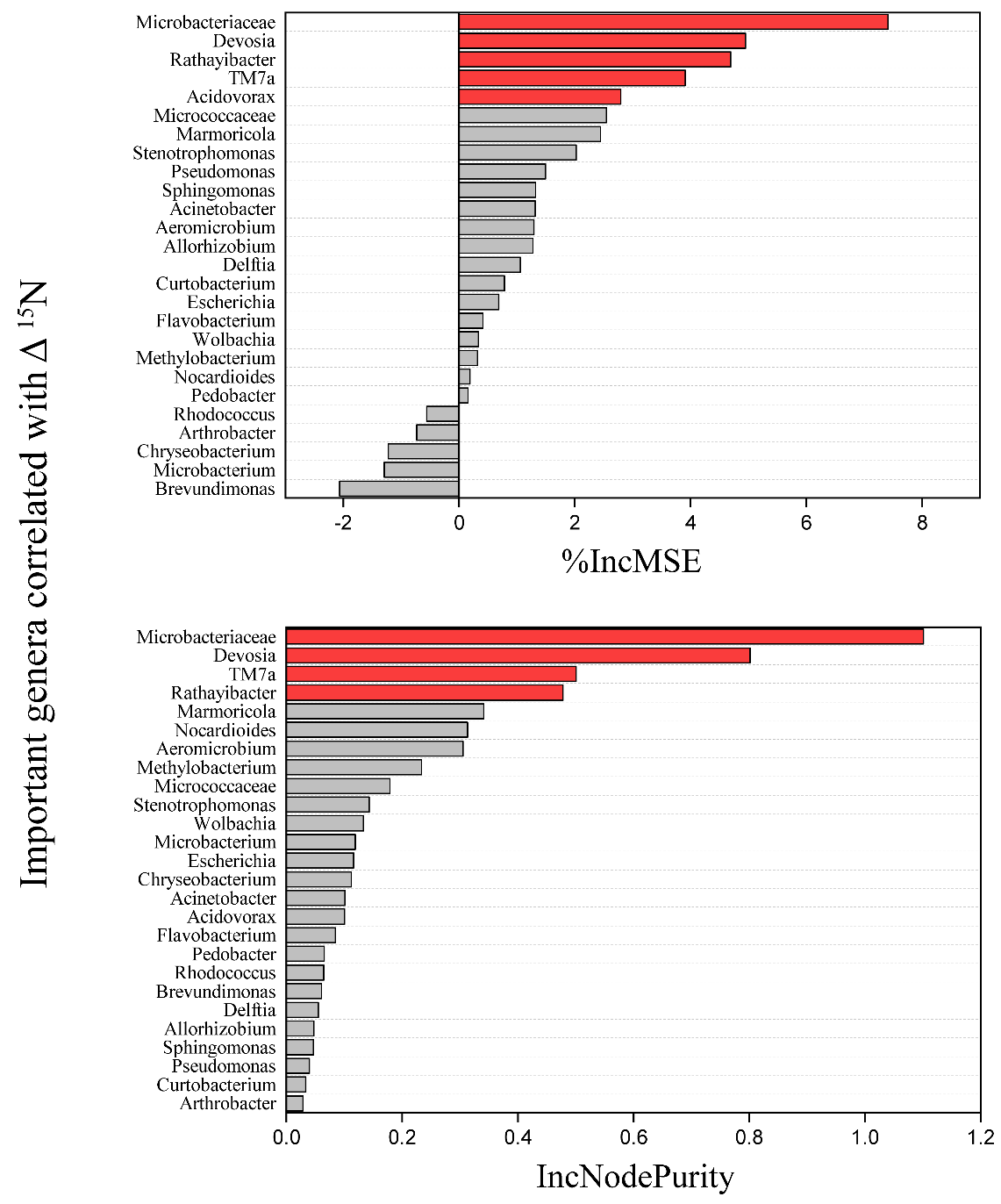


Supplementary Figure 6. Variable importance of the Random Forest regression model for the prediction of cold-adapted Collembola associated microbiota-Δ^15^N enrichment correlated with different food treatments. The variables show the bacterial genera with relative abundance > 1% in Collembola, ranked on the basis of their importance for the increase in the mean square error (MSE) and NodePurity. Genera with a red background indicate significantly important species (*p* < 0.05).

Supplementary Table 1 Values of δ^13^C and δ^15^N (mean ± standard error) in cold-adapted Collembola tissue and food.

|  |  | δ^13^C | δ^15^N |
| --- | --- | --- | --- |
| Food | Corn litter | -12.08 ± 0.02 a | -2.95 ± 0.03 b |
|  | Mongolian oak litter | -27.09 ± 0.02 c | -3.56 ± 0.03 c |
|  | Yeast | -15.65 ± 0.02 b | -1.64 ± 0.05 a |
| Collembola | Fed on corn litter | -16.97 ± 0.21 a | -0.07 ± 0.16 a |
|  | Fed on mongolian oak litter | -24.01 ± 0.13 c | -1.83 ± 0.54 b |
|  | Fed on yeast | -19.03 ± 0.35 b | 0.21 ± 0.10 a |

Different letters represent significant differences between different treatments (Tukey test, *p* < 0.05).

Supplementary Table 2 Pearson correlation between microbial genera (relative abundance > 1%) and stable isotopic enrichment in cold-adapted Collembola.

| Genus | Δ^13^C | | Δ^15^N | |
| --- | --- | --- | --- | --- |
|  | Correlated coefficient | *p* | Correlated coefficient | *p* |
| *Wolbachia* | 0.453 | *p*>0.05 | -0.530 | * |
| *Pseudomonas* | -0.077 | *p*>0.05 | -0.350 | *p*>0.05 |
| *Acinetobacter* | -0.207 | *p*>0.05 | 0.075 | *p*>0.05 |
| *Flavobacterium* | -0.242 | *p*>0.05 | -0.263 | *p*>0.05 |
| *Nocardioides* | 0.083 | *p*>0.05 | 0.551 | * |
| *Microbacterium* | -0.033 | *p*>0.05 | -0.148 | *p*>0.05 |
| *Sphingomonas* | -0.145 | *p*>0.05 | 0.303 | *p*>0.05 |
| *Chryseobacterium* | 0.254 | *p*>0.05 | 0.060 | *p*>0.05 |
| *Acidovorax* | -0.088 | *p*>0.05 | 0.023 | *p*>0.05 |
| *Aeromicrobium* | 0.395 | *p*>0.05 | 0.569 | * |
| *Escherichia* | -0.105 | *p*>0.05 | 0.215 | *p*>0.05 |
| *Delftia* | -0.012 | *p*>0.05 | -0.067 | *p*>0.05 |
| *Pedobacter* | -0.367 | *p*>0.05 | 0.018 | *p*>0.05 |
| *Allorhizobium* | 0.063 | *p*>0.05 | 0.009 | *p*>0.05 |
| *Brevundimonas* | -0.337 | *p*>0.05 | -0.142 | *p*>0.05 |
| *Marmoricola* | 0.191 | *p*>0.05 | 0.680 | ** |
| *Stenotrophomonas* | -0.183 | *p*>0.05 | -0.428 | *p*>0.05 |
| *Curtobacterium* | 0.463 | *p*>0.05 | 0.244 | *p*>0.05 |
| *Rhodococcus* | 0.203 | *p*>0.05 | 0.197 | *p*>0.05 |
| *Methylobacterium* | -0.152 | *p*>0.05 | 0.444 | *p*>0.05 |

** denotes *p* < 0.01; * denotes *p* < 0.05.
